# Mitochondrial DNA Copy Number and Developmental Origins of Health and Disease (DOHaD)

**DOI:** 10.3390/ijms22126634

**Published:** 2021-06-21

**Authors:** Hisanori Fukunaga

**Affiliations:** Center for Environmental and Health Sciences, Hokkaido University, N12 W7 Kita-ku, Sapporo 060-0812, Japan; hisanori.fukunaga.a1@cehs.hokudai.ac.jp

**Keywords:** DOHaD, environmental stress, mitochondrial DNA copy number, pregnancy, risk management

## Abstract

Mitochondrial dysfunction is known to contribute to mitochondrial diseases, as well as to a variety of aging-based pathologies. Mitochondria have their own genomes (mitochondrial DNA (mtDNA)) and the abnormalities, such as point mutations, deletions, and copy number variations, are involved in mitochondrial dysfunction. In recent years, several epidemiological studies and animal experiments have supported the Developmental Origin of Health and Disease (DOHaD) theory, which states that the environment during fetal life influences the predisposition to disease and the risk of morbidity in adulthood. Mitochondria play a central role in energy production, as well as in various cellular functions, such as apoptosis, lipid metabolism, and calcium metabolism. In terms of the DOHaD theory, mtDNA copy number may be a mediator of health and disease. This paper summarizes the results of recent epidemiological studies on the relationship between environmental factors and mtDNA copy number during pregnancy from the perspective of DOHaD theory. The results of these studies suggest a hypothesis that mtDNA copy number may reflect environmental influences during fetal life and possibly serve as a surrogate marker of health risks in adulthood.

## 1. Introduction

Since the recognition of mitochondrial diseases in humans in 1962 [1], numerous studies have elucidated the physiological and pathological significance of mitochondrial function. Mitochondria are intracellular organelles, which are present in almost every cell in the body and are responsible for the production of energy in the form of adenosine triphosphate (ATP) [2,3]. Thus, in patients with mitochondrial diseases, organs that are dependent on mitochondrial production for energy may be damaged [4]. In the neonatal period, a “triad” of cerebromuscular (e.g., recurrent apnea, inactivity, convulsions, and hypotonia), gastrointestinal and hepatic (e.g., vomiting, poor feeding, diarrhea, and hepatomegaly), and myocardial symptoms (e.g., arrhythmias, cardiomyopathy, and heart failure) related to mitochondrial diseases have been reported [5]. In infancy, symptoms of mitochondrial diseases include poor weight gain, progressive liver damage, cerebellar ataxia, pyramidal tract signs, psychomotor disturbances, respiratory failure, myotonia, and amino aciduria [6,7]. Central nervous system symptoms and muscular symptoms of mitochondrial diseases occur during childhood and adulthood. Three major mitochondrial encephalomyopathies have been reported: mitochondrial myopathy, encephalopathy, lactic acidosis, and stroke-like episodes [8,9,10]; myoclonic epilepsy and ragged-red fiber disease [11,12]; and chronic progressive external ophthalmoplegia, including Kearns–Sayre syndrome [13,14,15]. As the pathogenesis of mitochondrial disease suggests, the pathological significance of mitochondrial dysfunction is enormous. Mitochondrial dysfunction is also a hallmark of biological aging, as mitochondrial dysfunction worsens with age [16].

In eukaryotic cells, nuclear DNA (nDNA) and mitochondrial DNA (mtDNA) cooperate to regulate cellular functions [17]. Human mtDNA comprises a cyclic multicopy genome containing 16,569 base pairs, with usually tens to thousands of copies per cell. Human mtDNA is maternally inherited, as paternal mitochondria in fertilized eggs are removed by “mitophagy” [18,19]. Mitophagy, a quality control mechanism, is the selective degradation of mitochondria by autophagy for maintaining the health of the mitochondrial network [20,21]. Human mtDNA is independently replicated, and each genome mutates on its own, resulting in the formation of a condition called “heteroplasmy” [22,23]. Heteroplasmy means the existence of a normal version of the maternal mtDNA genome and a version of the genome containing a mutation, and this mixing rate easily changes as cells proliferate and differentiate. The mode of existence of human mtDNA also differs among tissues [24]. For example, mtDNA exists in single circles in many organs and cells, but in the heart, several mtDNA molecules are linked together in a complex network. The fact that the mode of existence of mtDNA differs in each tissue may be a consequence of the search for the most suitable state to maintain mtDNA in each tissue. While the mitochondrial genome is considerably smaller than that of the nuclear genome, the mRNA transcribed from mtDNA accounts for about 30% of total cellular mRNA in the heart and 5–25% of total cellular mRNA in other organs [25]. After being transcribed, mitochondrial RNA is processed, the mitoribosome is assembled, and respiratory chain proteins encoded by mtDNA are synthesized [26]. Human mtDNA at the population level is shaped by selective forces within the female germ line under nuclear genetic control [27]. According to the literature, the incidence of mitochondrial diseases is 9.6 per 100,000, of which 1.2 to 1.5 patients have a single mtDNA deletion [28,29,30]. The majority of mitochondrial diseases are inherited through autosomal or X-linked inheritance rather than maternal inheritance, and around 75% of mitochondrial diseases, especially those that occur in childhood, are caused by nDNA mutations [6,31]. Around 300 causative genes of human mitochondrial diseases have been identified using next-generation sequencing [32]. In an animal study, investigators removed the nucleus from a fertilized egg of a mouse with an mtDNA mutation and transplanted it into an unfertilized egg of a healthy female mouse [33]. In this study, the resulting litter was healthy. However, as mentioned above, such a technical approach would only be applicable to a subset of patients with mitochondrial diseases. In terms of the drug delivery system (DDS), which delivers target molecules to mitochondria, various attempts have been made to protect or activate mitochondrial functions [34]. Mitochondrial transplantation treatment [34,35] is one of these treatment challenges, but several issues remain to be addressed [35,36,37].

As shown in previous studies, the number of mtDNA copies in the peripheral blood decreases with age [38], and the decrease is negatively associated with age-related events, such as all-cause mortality [39]. In addition, mtDNA copy number loss is associated with cognitive function [40], cardiovascular diseases [41], and infectious morbidity and mortality in patients with chronic kidney disease [42]. Mitochondrial DNA depletion syndrome (MTDPS) refers to diseases in which the mtDNA copy number is reduced, and mitochondrial energy metabolism is impaired [43]. The underlying pathogenesis of MTDPS refers to abnormalities in genes related to the replication maintenance mechanism of mtDNA encoded in nDNA, resulting not only in mtDNA depletion but also in multiple deletions and point mutations [44]. These include abnormalities in *POLG*, *POLG2*, *Twinkle*, *RNASEH1*, *DNA2,* and *MGME1* genes [45]. The mode of inheritance of MTDPS is autosomal recessive [46]. As suggested by MTDPS associated with a variety of pathological conditions, dysregulation of mtDNA copy number and mitochondrial dysfunction play a role in a variety of pathologies. The maintenance of mtDNA copy number via mtDNA replication is of high importance not only at the cellular level but also at the individual level.

In terms of its physiological significance, mtDNA copy number is important from fetal, through infantile and childhood stages, to adulthood. In fact, mtDNA copy number influences early developmental cell differentiation and reprogramming of induced pluripotent stem cells [47]. In a previous study, the mtDNA contents of newborns with abnormal birth weights were lower than those of newborns with normal weights [48]. Based on accumulating evidence [49,50,51], reduced mtDNA content may be a putative link between abnormal fetal growth and metabolic and cardiovascular complications in later life. In a recent population-based follow-up study of middle-aged Swedish women (*N* = 2387), the mtDNA copy number was significantly lower both in women with type 2 diabetes and in those who developed type 2 diabetes during the follow-up period compared to those who did not develop type 2 diabetes [52]. Furthermore, a study based on data from the U.K. Biobank confirmed that a higher mtDNA copy number was significantly associated with a lower prevalence of neurodegenerative diseases (odds ratio (OR) = 0.89, confidence interval (CI) = 0.83; 0.96) and a lower incidence rate of neurodegenerative diseases (hazard ratio = 0.95, 95% CI = 0.91; 0.98) [53].

**Figure 1:**
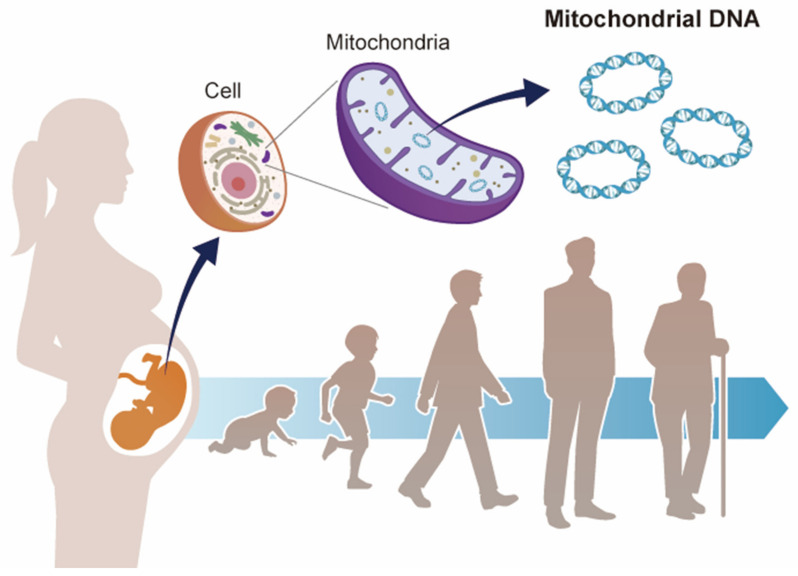
Mitochondrial DNA and developmental origins of health and disease (DOHaD). Mitochondrial DNA copy number increases or decreases in response to environmental changes. Environmental stress during fetal life may contribute to the risk of disease in adulthood via mitochondrial DNA copy number.

The Developmental Origin of Health and Disease (DOHaD)” theory posits that environmental factors during development act as risk factors for health and disease in adulthood [54,55,56,57,58,59,60]. Considering the physiological and pathological significance of mtDNA copy number from the perspective of DOHaD theory could shed light on the potential role of mtDNA copy number as a biomarker of disease in later life [61]. This paper discusses the potential of mtDNA copy number in early life as a biomarker of health and disease in later life (Figure 1), focusing on recent epidemiological studies that suggest a correlation between environmental factors and mtDNA copy number changes during the fetal period.

## 2. DOHaD Theory

The sum of chemical, physical, and biological stresses from the environment, namely the exposome, contributes to human health conditions [62]. Studies by Barker on infants with low birth weights in the 1980s [63,64,65] led to the development of the Fetal Origins of Adult Disease theory in 1990 [54]. Taking into account the results of subsequent epidemiological studies [66,67], the DOHaD theory was proposed in 2004 [55].

Today, a number of epidemiological studies and animal model studies suggest that environmental factors in embryonic, fetal, neonatal, and early childhood periods are associated with health and disease in adulthood and old age, including the risk of developing chronic diseases, supporting the DOHaD theory [68,69,70,71,72,73,74,75,76,77]. For example, in cases of food deprivation during pregnancy, the fetus acquires an energy-saving constitution in anticipation of the environment after birth, in which nutrient intake may be insufficient [78]. If the mother has a glucose-intolerant pregnancy (gestational diabetes mellitus (GDM)) or is obese, it can also cause obesity in the child. It is thought that the fetus receives an excess supply of glucose from the mother, which causes insulin to be overproduced. In addition, the growth-promoting effects of insulin and insulin-like growth factors (IGFs) may be the cause of obese children (gigantism). In addition to nutritional status, various environmental factors, such as exposure to environmental chemicals (e.g., endocrine-disrupting chemicals), are risk factors for disease development [79,80,81]. It is well known that passive smoke exposure during pregnancy interferes with fetal brain development [82]. Low birth weight, especially intrauterine growth retardation, is known to be a risk factor for chronic kidney disease and hypertension. The kidneys are made up of nephrons (renal units), and there are usually around 2 million nephrons per kidney. However, low maternal nutrition, infections, and drugs (ethanol, gentamicin, NSAIDs) can cause the number of nephrons in the fetus to be extremely low, which may lead to the development of hypertension and chronic kidney disease after birth [83]. Other factors such as delayed growth outside the uterus and postnatal administration of steroids and antimicrobials have also been suggested to be associated with the risk of decreased renal function, leading to renal disease. According to research, circadian rhythms during pregnancy also play a role in the health and well-being of children in adulthood, including their risk of particular diseases [84,85]. In addition, gestational hypertension and gestational diabetes may lead to worse health outcomes in newborns [86,87]. Previous studies have provided support for the roles of both the epigenome and gut microbiota during the early postnatal period in future health, thereby supporting the DOHaD theory [88,89,90,91].

Based on the DOHaD theory, there is believed to be a link between exposure to external stress in the fetal environment and risk of noncommunicable diseases (NCDs) in adulthood [92]. Given the increase in the proportion of the aging population in many developed countries, reducing health expenditure through the prevention of NCDs has become an important social and political issue. Previous research suggested that research focusing on the DOHaD was important to develop regional strategies to contribute to a reduction in the incidence of NCDs in low-income countries [93].

## 3. Mitochondrial DNA Copy Number

In 1988, human mitochondrial transcription factor A (TFAM) was originally identified as a transcription factor for mtDNA [94]. Later, it was found that an important role of TFAM is to stabilize mtDNA by binding to the entire mtDNA region in a sequence-nonspecific manner [95]. Today, TFAM is known to be a major protein in the mtDNA–protein complex called mitochondrial nucleoid [96]. Since the expression level of TFAM in cells correlates with the amount of mtDNA [97], it has been proposed that mtDNA can exist stably depending on the amount of higher-order structures that can be formed in TFAM. In addition, Lon protease is thought to be indirectly involved in the regulation of mtDNA content because it specifically degrades TFAM [98].

*TWNK* mutant mice are known as an animal model for reduced mtDNA copy number [99]. The *TWNK* gen encodes Twinkle, a helicase that unwinds the mtDNA double helix and plays an important role in mtDNA replication. In these mice, there is a significant decrease in tissue respiratory function and central nervous system mtDNA copy number after 12 months of age. This suggests that *TWNK* gene mutations may be involved in the pathogenesis of MTDPS. In the future, further establishment and detailed analysis of animal models of mitochondrial diseases are necessary to understand the complex pathogenesis of mitochondrial diseases and the regulatory mechanism of mtDNA copy number.

It is well known that mitochondria are targets of environmental compounds, such as pesticides, polychlorinated biphenyls, heavy metals, and particulate matters (PMs) [100]. In a study on polycyclic aromatic hydrocarbons (PAHs), a widespread environmental pollutant produced during the combustion process, the blood mtDNA content decreased by 9.85% (95% CI: −15.16 to −4.2; *p* = 0.002) for every doubling of nonvolatile PAH content in house dust during winter, regardless of sex, age, body mass index, or grilled meat/fish consumption (0.002) [101]. The corresponding estimate for volatile PAHs was −7.3% (95% CI: −13.71 to −0.42; *p* = 0.04). A study that examined the relationship between nonoccupational exposure to PM_2.5_, benzo[a]pyrene (BaP), a PAH, and mtDNA copy number found an inverse relationship between mtDNA copy number and exposure to PM_2.5_ and BaP [102]. Every 1 log-μg/m3 increase in PM_2.5_ was associated with significant mtDNA copy number loss (−10.3 copies per cell) (95% CI: −18.6, −2.0, *p* = 0.02). Occupational exposure to PM via dust also caused changes in mtDNA copy number [103]. The results of the Beijing Truck Driver Air Pollution Study on 60 truck drivers and 60 office workers just prior to the 2008 Beijing Olympics (15 June to 27 July 2008) observed a decrease in blood mtDNA copy numbers in association with increased exposure to elemental carbon during working hours and recent exposure to PM10 in the air [104].

In general, the mtDNA copy number is calculated from the amplification products of genes in the mitochondrial genome using a quantitative real-time polymerase chain reaction (PCR) assay, with the amplification products of genes in the nuclear genome used as a reference [105]. A 2021 study by the Genotype-Tissue Expression Project examined the relationship between whole-blood mtDNA copy numbers in 419 individuals and peripheral blood and tissue gene expression [53]. In the study, the mtDNA copy number was significantly associated with the expression of 700 genes, including nuclear genes required for mtDNA replication. This indicates that the mtDNA copy number in peripheral blood would be a biomarker that reflects the metabolic status of multiple tissues. In the future, it will be necessary to accumulate knowledge to support the usefulness of mtDNA copy number in peripheral blood as a biomarker.

## 4. Environmental Factors during Pregnancy and mtDNA Copy Number in Newborns

The oocyte mtDNA copy number is tightly regulated during development [106]. Primordial germ cells have a low amount of mtDNA, about 200 copies per cell. These copies form the template for all mtDNA transmitted through the germline and are replicated exponentially during oogenesis. A mature egg has the highest mtDNA copy number of any cell. This high content is directly related to the capacity of the oocyte to support the early stages of embryonic development in many species [107]. Epigenetic reprogramming depends on the metabolic cofactors produced by the mitochondrial metabolism, and the reactive oxygen species derived from the mitochondrial respiratory chain are essential for the regulation of cell signaling in the embryo. Therefore, if the environment reduces the oocyte mtDNA copy number, it would be expected to affect not only the bioactivity of the oocyte but also the health of the fetus.

In the context of the DOHaD theory, several epidemiological studies have suggested that maternal exposure to environmental chemicals during pregnancy may be involved in changes in fetal mtDNA copy number. In this regard, many studies have focused on the correlation between air pollution and cord blood mtDNA copy number [108]. As mtDNA is susceptible to environmental toxicants due to its limited repair capacity [109], mitochondrial dysfunction via abnormalities in mtDNA can be caused by air pollution. Mitochondrial dysfunction in the placenta may contribute to growth restriction of the fetus by limiting intracellular energy utilization. Oxidative damage, inflammation, and hypermethylation have been proposed as the main mechanisms underlying mtDNA dysfunction, with a correlation between DNA methylation and mtDNA replication, both of which are known to regulate cell fate during development [17].

It has been well documented that exposure to airborne particles and passive smoke exposure during pregnancy can affect birth weights [110,111,112]. In 2005, Bouhours-Nouet et al. reported a 37% decrease in the relative content of mtDNA in placental tissue from smokers compared to that in nonsmokers (*p* < 0.02) [113]. A 2012 study reported that a 10 µg/m^3^ increase in exposure to PM_10_ in the last month of pregnancy resulted in a 16.1% decrease in placental mtDNA content (95% CI: −25.2, −6.0%, *p* = 0.003) [114]. Furthermore, the same group reported in 2015 that an increase in the interquartile range for PM_2.5_ exposure during pregnancy was positively correlated with mtDNA methylation in placental tissue (*p* = 0.05) and inversely correlated with mtDNA content (relative change: −15.60%, *p* = 0.001) [115]. According to a study in 2020, prenatal PM_2.5_ exposure at 25–32 weeks and PM_10_ exposure at 25–31 weeks were significantly associated with a decrease in cord blood mtDNA copy number [116]. PM_2.5_ exposure in the third trimester was also associated with mtDNA copy number loss (cumulative change: −8.55%, 95% CI: −13.32%, −3.51%). On the other hand, a 2018 study reported that PM_10_ exposure in early pregnancy was associated with mtDNA copy number gain during pregnancy [117].

In 2016, Clemente et al. reported a correlation between environmental NO_2_ exposure and a reduction in cord blood mtDNA copy number during pregnancy in two independent cohort studies in Spain and Belgium, suggesting a correlation between cord blood mtDNA copy number loss and low birth weight [118]. Furthermore, they reported that NO_2_ exposure during pregnancy is associated with low birth weight via a reduction in cord blood mtDNA [119].

Maternal exposure to metals also affects cord blood mtDNA copy number. For example, a study reported a correlation between thallium exposure during pregnancy and a decrease in cord blood mtDNA copy number [120], and arsenic exposure during pregnancy and a decrease in cord blood mtDNA copy number [121]. On the other hand, aluminum exposure was associated with an increase in cord blood mtDNA copy number [122].

Exposure to PAHs during pregnancy is a risk factor for adverse neurobehavioral development. According to the literature in 2020, prenatal exposure to PAHs decreases neurobehavioral development scores and increases mtDNA copy number in children [123]. In addition, PAH exposure in utero is linked to neurobehavioral development disorders in children. Exposure to benzotriazoles and benzothiazoles can interfere with mitochondrial function by inducing oxidative stress, which may contribute to adverse health effects. In fact, a 2020 study reported that prenatal exposure to benzotriazoles and benzothiazoles is associated with changes in cord blood mtDNA copy number and that the effects varied according to the sex of the infant [124]. In a study on bisphenol S (BPS) commonly used as a substitute for bisphenol A, BPS exposure in male newborns is associated with a reduction in cord blood mtDNA copy number [125].

Psychosocial stress is known to contribute to oxidative stress in the placenta [126]. The Programming of Intergenerational Stress Mechanisms study examined the association of maternal lifetime stress, negative life events, depression, and post-traumatic stress disorder symptom scores with placental mtDNA copy number [127]. In a linear regression analysis, adjusting for maternal age, race/ethnicity, education, prenatal exposure to fine PM, prenatal exposure to smoking, and child sex, all stress measures were associated with placental mtDNA copy number loss (all *p* values < 0.05). These results were the first to point to an association between maternal psychosocial stress and placental mtDNA copy number loss.

In 2018, Priliani et al. examined the association between maternal mitochondrial function, as expressed by the mtDNA copy number in venous blood, and birth weights in 528 randomly selected mothers [128]. A real-time quantitative PCR assay of archived blood samples and a regression analysis, adjusting for other major determinants of birth weight, showed that their mtDNA copy numbers were inversely correlated with birth weight (*p* < 0.001), especially in the third trimester (*p* < 0.001). Furthermore, they showed that maternal multiple micronutrient supplementation improved the mtDNA copy number in pregnant women [129]. Although the turnover and mechanism of mtDNA synthesis in maternal blood, cord blood, and placental tissue may be different [130], based on the literature, a nutritional approach may mitigate the health effects of environmental chemical exposure through changes in the mtDNA copy number.

These results suggest that the copy number of mitochondrial DNA in maternal blood or cord blood increases or decreases in response to environmental changes during pregnancy. Although the mechanism is not fully understood, the recent accumulation of epidemiological findings leaves no doubt about the causal relationship, and scientific evidence is accumulating to support the DOHaD theory. It may be acceptable to regard mitochondrial DNA copy number as a kind of DOHaD mediator. However, in the future, it will be necessary to clarify which part of the mechanism controlling mitochondrial DNA copy number can be disrupted by environmental stress.

## 5. Conclusions

This paper summarizes the results of recent epidemiological studies on maternal exposure to various environmental factors during pregnancy and mtDNA copy number loss and gain from the perspective of the DOHaD theory (Table 1). Our understanding of environmental stress during pregnancy and changes in cord blood mtDNA copy number has increased, especially in epidemiological studies of air pollution. Susceptibility to PMs seems to vary depending on the time of pregnancy. While it remains debatable, human mtDNA copy number would be an indicator of environmental influences during fetal life, as well as a surrogate marker of disease risk in adulthood. Thus, here, the author proposes a hypothesis that mtDNA copy number may be the “missing link” between temporally distant events, such as environmental exposures during development and morbidity risk in adulthood. In the future, the preservation of mtDNA copy number during the developmental period will lead to novel disease-preventive strategies and therapies. For example, further investigation aimed at developing NCD prevention strategies could focus on interventions, such as nutritional supplementation, in high-risk groups during the pre- and perinatal periods. 

## Figures and Tables

**Table 1:** Recent epidemiological studies on environmental factors during pregnancy and cord blood mtDNA copy number.

Environmental Factor	Exposure	Cord Blood mtDNA Copy Number	Reference
Air pollution	Smoking	Decrease	[113]
	PM_10_ (last month)	Decrease	[114]
	PM_2.5_	Decrease	[115]
	PM_2.5_ (25–32 weeks) and PM_10_ (25–31 weeks)	Decrease	[116]
	PM_10_ (early pregnancy)	Increase	[117]
	NO_2_	Decrease	[118,119]
Metal	Thallium	Decrease	[120]
	Arsenic	Decrease	[121]
	Aluminum	Increase	[122]
Environmental chemical	PAHs	Decrease	[123]
	Benzotriazoles and Benzothiazoles	Decrease	[124]
	BPS	Decrease	[125]
Psychosocial stress	Maternal lifetime stress, Negative life events, depression, and post-traumatic stress disorder	Decrease	[127]
